# Nonlinear Optical Response of Gold Nanobipyramids for a Doubly Q-Switched Ho-Doped Laser at a Wavelength of 2.1 µm

**DOI:** 10.3390/nano11020535

**Published:** 2021-02-19

**Authors:** Cheng Zhang, Dongzhou Wang, Shengjun Huang, Jimin Yang, Jie Liu, Jing Fang

**Affiliations:** 1 Shandong Provincial Engineering and Technical Center of Light Manipulations and Shandong Provincial Key Laboratory of Optics and Photonic Device, School of Physics and Electronics, Shandong Normal University, Jinan 250358, China; 13256715081@163.com (C.Z.); 2020020534@stu.sdnu.edu.cn (S.H.); jmyang@sdnu.edu.cn (J.Y.); 2 Jinan Institute of Quantum Technology, Jinan 250101, China; wangdongzhou@jiqt.org

**Keywords:** gold nanobipyramids, saturable absorber, doubly Q-switched, Ho-doped laser, 2.1 μm

## Abstract

Gold nanobipyramids (Au-NBPs) were successfully fabricated using the seed-mediated growth method. The saturable absorption performance of the Au-NBPs at a 2-μm band wavelength was characterized. Using excellent-quality, mature Ho:YLF crystals, a doubly Q-switched (DQS) laser joining an acousto-optic modulator (AOM) with an Au-NBP saturable absorber (SA) was achieved. When the modulation rate of the AOM was 1 kHz, the shortest pulse width (54 ns) was attained, corresponding to the highest peak power (3.87 kW). This was compared with a singly Q-switched laser joining an AOM with an Au-NBP SA, whereby the maximum pulse width compression ratio was 15.2 and the highest peak power enhancement factor was 541.3. Our study has shown that Au-NBPs are a potential saturable absorption nanomaterial, and the DQS laser has the benefit of compressing the pulse width and increasing the peak power at a wavelength of 2.1 μm.

## 1. Introduction

Ho-doped pulsed lasers, with a narrow pulse width, high repetition rate, and good symmetry, have wide applicability in the fields of micro-machining, ranging, remote sensing, microsurgery, etc. [1,2,3]. Particularly for highly symmetrical pulses, energy can be concentrated on the soft edge of the pulse without wastage [4,5,6]. Q-switching is an effective method for deriving a short pulse mode, and includes two well-known traditional techniques—active Q-switching (AQS) and passive Q-switching (PQS) [7,8]. AQS lasers have a controllable frequency, high peak power, and stable pulse operation [9,10,11,12]. However, AQS lasers’ pulse temporal profiles tend to be asymmetric, with a sharply rising edge and a slowly falling edge. Fortunately, PQS lasers with saturable absorbers (SAs) have a slowly rising edge and a rapidly falling edge, and can effectively complement the AQS’s pulse temporal profile. Moreover, PQS operation has attracted much attention from researchers because of the inherent compactness, simplicity, and low cost of their cavities [13,14,15,16,17,18,19]. In recent years, the emergence of low-dimensional nanomaterials with saturable absorption characteristics has become one of the major driving factors of PQS operation [20,21,22]. Noble metal nanomaterials have attracted wide interest as a result of their relevance to localized surface plasmon resonance (LSPR). LSPR is excited at the interface of the free-electrons and the incident light field near the metal’s surface. The electro-magnetic resonances are associated with the particle size and shape, as well as the refractive index of the surroundings [23]. This therefore provides a way to control light confinement at a nanoscale, and opens up the possibility of extensive applicability, such as in surface-enhanced Raman spectroscopy, biological sensing, ultrafast information processors, and cancer diagnostics and therapy [24,25,26,27]. More importantly, plasmonic effects can enhance the nonlinear optical response, which plays a key role in optical modulation [28,29,30]. For gold nanoparticles, the third-order optical nonlinear effect is the most prominent, which enables them to act as SAs for all-optical switching and for the modulation of light. For example, the Teri Odom group successfully used gold nanoparticles in nanoscale plasma lasers [31]. Compared with other shapes of gold nanoparticles (e.g., rods and spheres), gold nanobipyramids (Au-NBPs) offer a unique structure with two tips, and possess the advantages of large local field enhancements, large extinction cross-sections, adjustable surface wavelengths, and high figures of merit [32,33]. Au-NBPs have a large third-order nonlinear optical susceptibility of 3.9 × 10^−13^ esu, as well as an ultrafast optical response time of ∼83 s at the wavelength of the longitudinal surface plasmon resonance (LSPR), due to their large local field enhancements [34]. As far as we are aware, the use of Au-NBPs as SAs in Ho-doped pulsed lasers at a wavelength of 2.1 µm has not been investigated.

A doubly Q-switched (DQS) laser with an acousto-optic modulator (AOM) and SA can simultaneously compress the pulse width, improve the pulse symmetry, and enhance the peak power. This kind of pulsed laser has multiple functions in different applications. For example, it can be directly used as the initial pulse after amplification, without any reshaping. In the past few years, some relevant studies have reported on its use in the 2-μm wavelength region. In 2015, Luan et al. constructed a DQS Tm:LuAG laser with an AOM and a graphene SA [35]. The maximum pulse width compression ratio here was found to be 3.11, and the highest peak power was enhanced 97.4 times. In 2019, Niu et al. demonstrated a DQS Tm:YAP laser with an AOM and a g-C_3_N_4_ SA, which had a minimum pulse width of 239 ns and a maximum peak power of 1146 W [36]. In 2020, Gao et al. reported on a DQS Tm:Ca(Gd,Lu)AlO_4_ laser using an AOM with a WS_2_ SA, through which the shortest pulse width of 91 ns and the highest peak power of 1.2 kW were obtained [37]. In the same year, Niu et al. presented a DQS Tm:Ca(Gd,Lu)AlO_4_ laser using an AOM and an MoS_2_ SA. It was reported that the maximum pulse compression ratio was 9.85 and the highest peak power enhancement factor was 123 [38]. To our knowledge, there are, as yet, no reports on DQS operation in a Ho-doped laser using an AOM and a nanomaterial SA at a wavelength of 2.1 µm.

In this paper, an Au-NBP SA was fabricated using the seed-mediated growth method, and the nonlinear absorbed performance was characterized. By using an AOM and an Au-NBP SA, a DQS Ho-doped laser operating at a 2.1 μm wavelength was achieved. When the absorbed pump power was 1.36 W, a minimum pulse width of 54 ns and a maximum peak power of 3.87 kW were obtained. In comparison with the singly Q-switched operation, the DQS laser achieved a shorter pulse width and a higher peak power.

## 2. Fabrication and Characteristics of the Au-NBP SA

**Figure 1:**
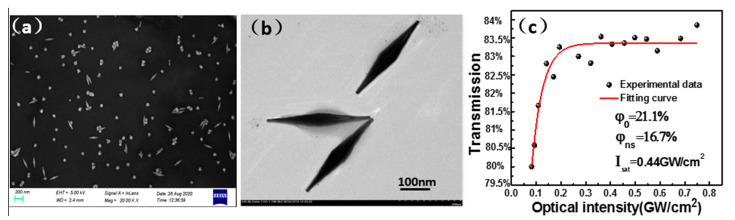
(**a**) Scanning electron microscope (SEM) image, (**b**) transmission electron microscope (TEM) image, and (**c**) nonlinear transmission curve.

The preparation of the Au-NBPs was completed at the start of this experiment. In the first step, we combined 50 µL of chloroauric acid and 74 µL of sodium citrate in a beaker with deionized water, and stirred for a few minutes. After the two solutions had fully reacted, 150 µL sodium borohydride was added and slightly stirred to complete the preparation of the gold seeds. In the second step, 40 mL of cetyltributylammonium bromide, 2 mL of chloroauric acid, 400 µL of silver nitrate, and 800 µL of ascorbic acid were added to the beaker and stirred, in order to prepare the growth solution. Finally, we used a plastic dropper to drop 20 mL of gold seeds into the growth solution, which was stirred with a glass rod and put into a centrifuge for centrifugation. After centrifugation, we poured the mixture into a closed glass bottle (to prevent it from drying) and kept it there for several hours to obtain the Au-NBP solution. The final Au-NBP SA film was formed by casting the dispersion onto a flat quartz substrate, followed by slow-drying at room temperature. As a background experiment, the transmission of the blank quartz substrate was also measured under the same conditions, and no nonlinear absorption behavior was observed. A scanning electron microscope (SEM) image of the Au-NBP SA film is shown in Figure 1a. The Au-NBPs were evenly distributed on the substrate. A transmission electron microscope (TEM) image of the Au-NBP SA film is shown in Figure 1b, at a scale bar of 100 nm. The structure of the Au-NBPs can be seen to extend along the base to the two ends, with two sharp vertices, which together generate a stronger local surface plasmon resonance electromagnetic field. The nonlinear saturable absorption properties of the Au-NBPs were calculated using a mode-locked fiber laser, with a 23.6 ps pulse duration and a 31 MHz repetition rate at 2000 nm. We measured the transmission by changing the intensity of the laser seed source power (Figure 1c). The calculation formula is shown below [19]:

(1) $$T\left(I\right)=1-\varphi_{0}*\exp\left(-\frac{I}{I_{sat}}\right)-\varphi_{ns}$$

The modulation depth ($\varphi_{0}$) of the Au-NBP SA was calculated to be about 21.1%, as shown in Figure 1c. The non-saturable absorption loss ($\varphi_{ns}$) and saturated light intensity ($I_{sat}$) were measured to be around 16.7% and 0.044 GW/cm^2^, respectively.

## 3. Experimental Setup

**Figure 2:**
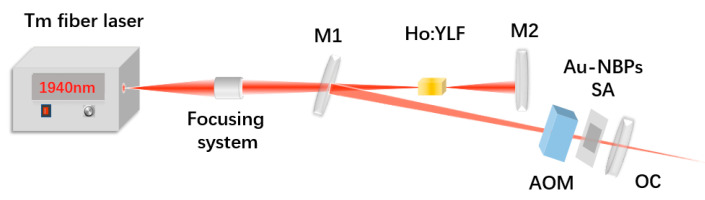
The schematic of the experimental setup for the doubly Q-switched (DQS) operation.

The experimental setup for characterizing the Ho:YLF DQS laser operation is shown in Figure 2. A commercial Tm:fiber laser (TDFL01-00015) with a maximum power of 30 W was used as the pump source. The central wavelength of the Tm:fiber laser was 1940 nm and the temperature was maintained at 22 °C. In order to prevent the pumped laser from returning and destroying the Tm:fiber laser, we adopted a simple V-shaped plane-concave cavity with a physical length of 196 mm. A lens with a focal length of 100 mm was used to collimate and focus the pump laser into the crystal. The block-shaped Ho:YLF crystal had a dopant concentration of 0.5 at. %, and dimensions of 3 × 3 × 10 mm^3^. The faces on either end were antireflection-coated at 1940 nm and 2050 nm. To increase the heat dissipation, the Ho:YLF crystal was wrapped in indium foil and tightly fastened in a water-cooled Cu billet, with the cooling water’s temperature (accuracy of 0.1 °C) kept stable at 13.0 °C. The laser resonator consisted of a flat pumping mirror (M1; 1850–1950 nm, T > 99%; 2050–2150 nm, R > 99.5%); a flat high-reflective mirror (M2) with identical parameters; and a plane-concave output coupler (OC) at 2050–2150 nm, with a 200 mm radius of curvature and partial transmissions (T) of 3%. The Au-NBP plate was located 5 mm from the OC in the resonator, functioning as the SA. An AOM of 52 mm in length was placed between the M1 and OC, acting as an active Q-switch. The AQS material was crystalline quartz, with a high transmission of under 2 μm operation. Using the famous ABCD matrix to calculate the beam radius in the cavity of the Ho:YLF laser, the corresponding cavity |A + D|/2 value was assessed to be less than 0.5, indicating that the cavity would always be stable.

## 4. Results and Discussions

**Figure 3:**
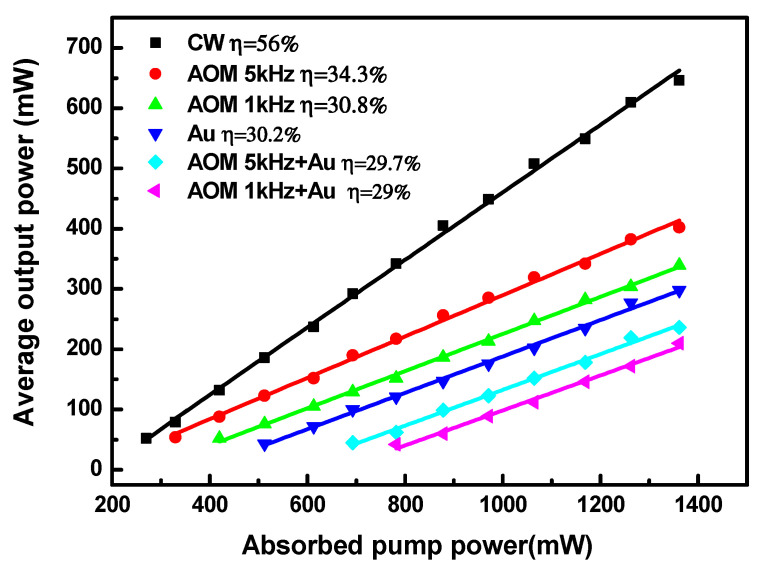
Average output powers of continuous wave (CW) and Q-switched lasers versus the absorbed pump powers.

A DQS laser was constructed by inserting two modulation devices. In addition, by removing the AOM, a singly passive (PQS) laser was obtained, and by removing the Au-NBP SA, an AQS laser was obtained. Without the AOM or Au-NBP SA in the cavity, continuous wave (CW) operations could be achieved. In this experiment, we controlled the modulation rate (MR) of the AOM at 1 and 5 kHz. Figure 3 displays the average output powers as a function of the absorbed pump power. It can be seen that the output powers increased approximately linearly as the absorption pump power increased. During the whole experiment, because of the low absorption, the HO:YLF crystal was not damaged when the injection pump’s power was increased to its highest level. When the absorption pump power reached 270 mW, the CW laser was the first to commence operation. Immediately afterwards, when the absorption pump power levels were 330, 420, 215, 693, and 782 mW, the AQS (at MRs of 5 and 1 kHz), PQS, and DQS (at MRs of 5 and 1 kHz) pulsed lasers started to work, respectively. At an absorption pump power of 1.36 W, average output powers of 646, 298, 339, 402, 210, and 236 mW were achieved for the CW, PQS, AQS (at MRs of 1 and 5 kHz), and DQS (at MRs of 1 and 5 kHz) lasers, respectively. For the AQS laser, an MR of 5 kHz offered better utilization of the population inversion of the Ho:YLF crystal compared with an MR of 1 kHz; however, both exhibited a higher output power than the PQS laser. 

**Figure 4:**
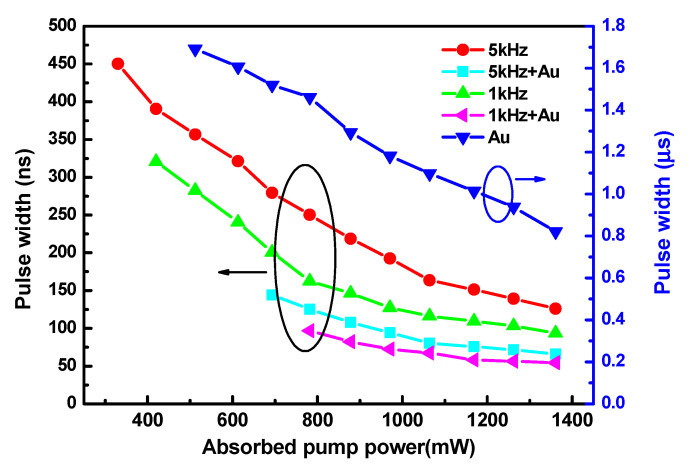
Pulse widths of Q-switched lasers versus the absorbed pump powers.

The pulse widths of the Q-switched lasers are displayed in Figure 4. It can be seen from the curve that the pulse widths of different Q-switched lasers decreased with increases in the absorbed pump power, and the pulse widths generated by the DQS lasers were shorter than those of the singly Q-switched lasers. The shortest pulse widths of 821, 94, 126, 54, and 66 ns for PQS, AQS (at MRs of 1 and 5 kHz), and DQS (MR of 1 kHz; 5 kHz), respectively, were acquired at the highest absorbed pump power. In order to better illustrate the comparison of the pulse widths, the compression ratio of the pulse width was invoked, which is defined as follows:

(2) $$t_{c}=t_{s}/t_{d}$$

where $t_{s}$ and $t_{d}$ are the pulse widths of the singly and DQS lasers, respectively. Regarding the AQS laser, the compression ratios ($t_{c}$) were 1.74 and 1.91 at 1 and 5 kHz, respectively, under the absorbed pump power of 1.36 W, and the compression ratios ($t_{c}$) were 15.2 and 12.44, in comparison with the PQS laser.

**Figure 5:**
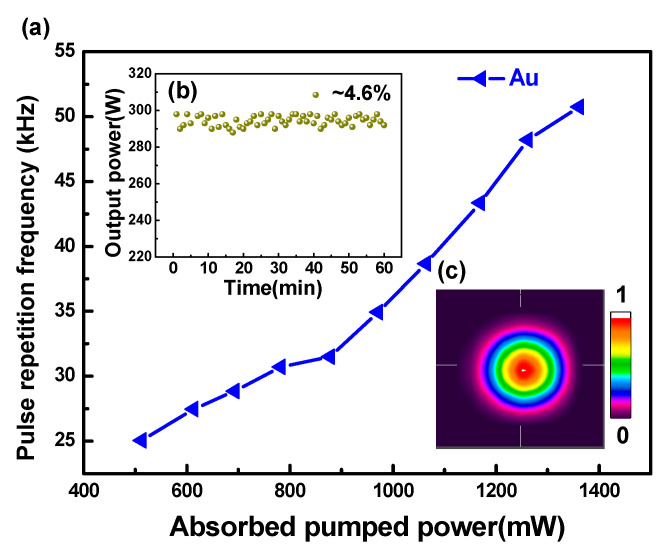
(**a**) Pulse repetition frequency of the passive Q-switched (PQS) laser with gold nanobipyramids (Au-NBPs) versus absorbed pump power. (**b**) Instability of the average output power measured over 60 min. (**c**) Spatial beam profile of the PQS laser.

**Figure 6:**
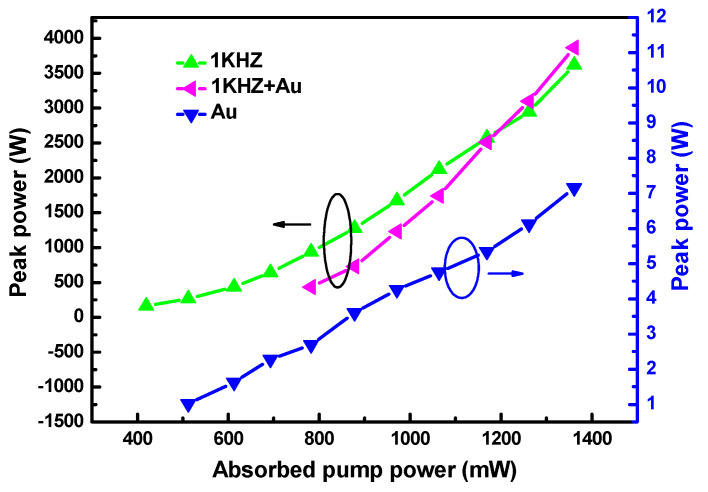
Peak powers of the PQS laser, as well as active Q-switched (AQS) and doubly Q-switched lasers at an acousto-optic modulator (AOM) repetition rate of 1 kHz, versus the absorbed pump power.

**Figure 7:**
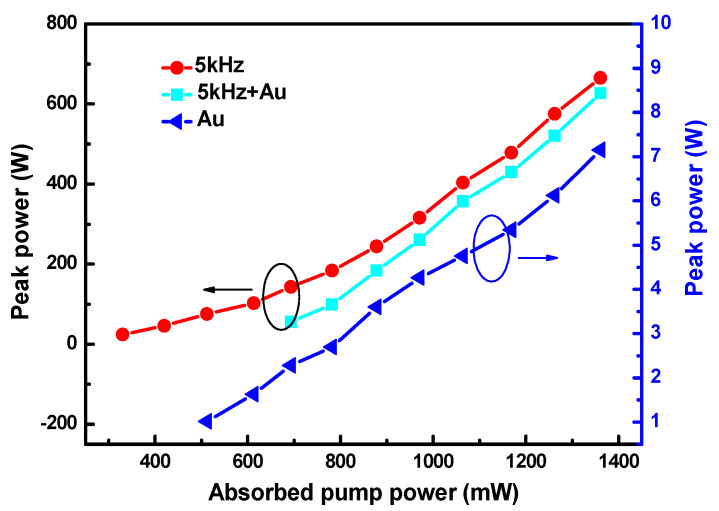
Peak powers of the PQS laser, as well as AQS and DQS lasers at an AOM repetition rate of 5 kHz, versus the absorbed pump power.

Another important characteristic of pulsed lasers is repetition frequency. For the singly PQS laser with Au-NBPs, the pulse repetition rate (PRR) gradually increased from 25.05 kHz to 50.74 kHz as the absorbed pump power increased (Figure 5a). For the AQS and DQS lasers, the PRRs were equal to the modulation rate of the AOM. Where the insertion shows the fluctuation of the maximum output power over an hour, we calculated an instability of about 4.6% (Figure 5b). The outline of the laser beam was recorded using an NS2-Pyro/9/5-PRO (Photon) instrument, as shown in Figure 5c. The figure shows that the shape of the spot is approximately circular. Different colors represent the light intensity distribution, which is symmetrical and conforms to the standard of the TEM_00_ mode. According to the repetition frequency and average output powers given above, the pulse peak powers were calculated, and are shown in Figure 6 and Figure 7. As can be seen in the figure, the peak powers acquired with the DQS laser were higher than those acquired with the singly Q-switched laser. At the highest absorbed pump power, the peak powers of PQS, AQS (at MRs of 1 and 5 kHz), and DQS (at MRs of 1 and 5 kHz) were 7.15, 3615, 3870, 638, and 714 W, respectively. An enhancement factor of $P_{i}$ was also used, as defined below:

(3) $$P_{i}=P_{d}/P_{s}$$

where $P_{s}$ and $P_{d}$ represent the pulse peak powers of the singly Q-switched and DQS lasers, respectively. For the singly AQS laser using an AOM, when the MRs were set at 1 and 5 kHz, the values of $P_{i}$ were 1.07 and 1.12, respectively. However, for the PQS laser using Au-NBPs with an AOM of 1 and 5 kHz, the $P_{i}$ values were 541.30 and 99.86, respectively. The results indicate that the peak powers of the DQS lasers were greatly improved.

**Figure 8:**
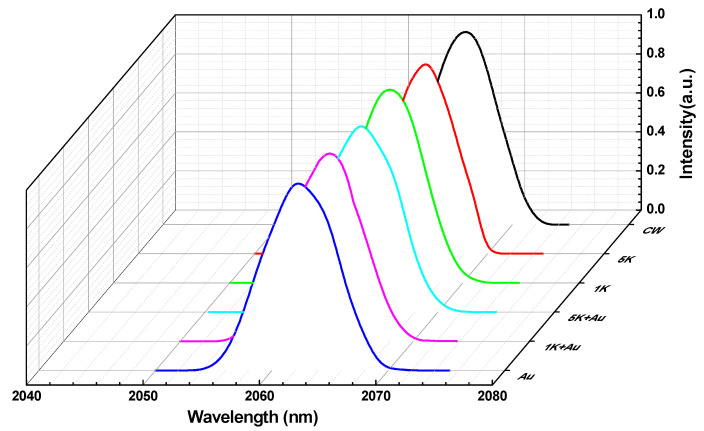
Output spectra of the CW, AQS (at 5 and 1 kHz), PQS, and DQS (at 5 and 1 kHz) lasers.

The output spectra of the CW and Q-switched operations are shown in Figure 8. The central wavelength of the laser was located around 2065.86 nm in the CW mode, while it shifted to 2061.9, 2063.81, and 2064.87 nm for the singly Q-switched laser with the Au-NBPs and AOMs (at MRs of 1 and 5 kHz), respectively. Output wavelengths around 2062.89 and 2063.41 nm were observed with the DQS laser using AOMs (at MRs of 1 and 5 kHz), respectively. These spectra may result from the mutual, beneficial interactions of the laser crystal, the working wavelength of the SA, the longitudinal mode frequency condition of the cavity, and the loss condition.

**Figure 9:**
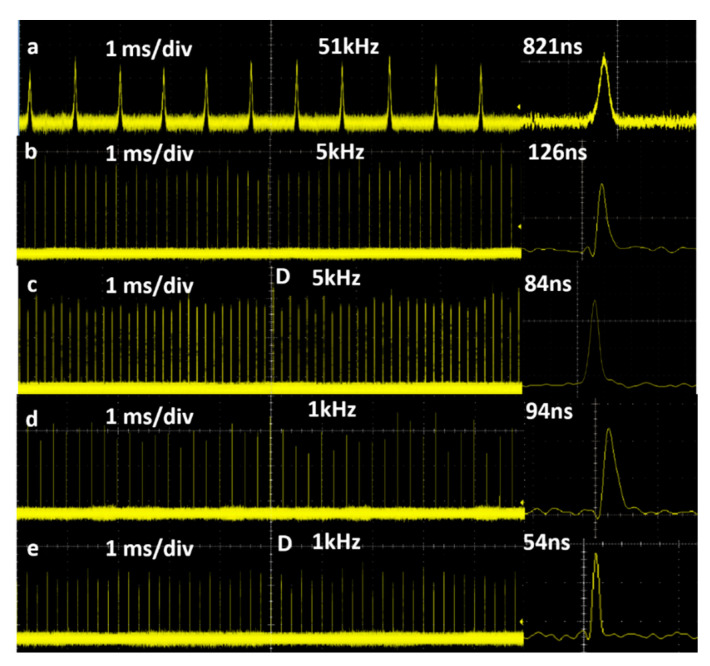
Temporal traces of pulse trains: (**a**) Au-NBPs PQS, (**b**) AQS at 5 kHz, (**c**) DQS at 5 kHz, (**d**) AQS at 1 kHz, and (**e**) DQS at 1 kHz.

Figure 9 shows the temporal Q-switched pulse profiles under the highest absorbed pump power of 1.36 W. There was a large inter-pulse fluctuation in the PQS laser, and the pulse interval was unstable. These phenomena were ameliorated in the AQS laser, and were further amelioration in the DQS laser, where the PRRs were controlled by the AOM and the fluctuations between pulses were slight.

**Figure 10:**
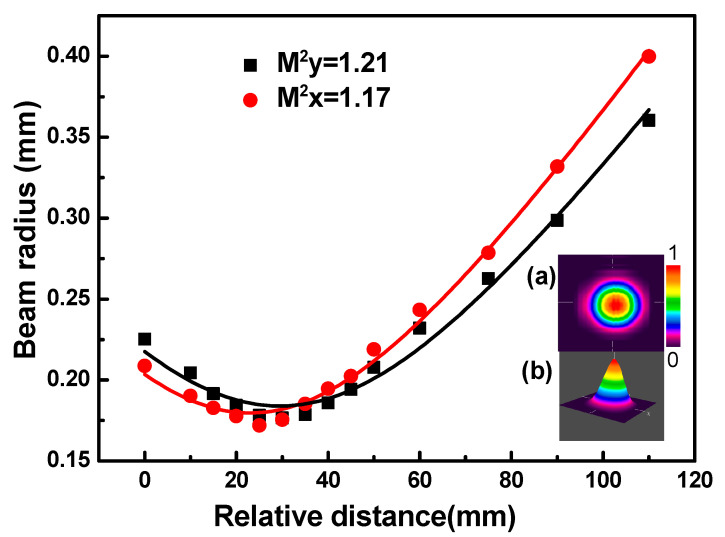
Beam quality (M^2^) factors: (**a**) spatial beam profile of the PQS laser and (**b**) 3D light intensity distribution of the DQS laser.

The scanning knife-edge method was used to measure the beam quality factor (M^2^). After passing through the focusing lens, the beam radii of the pulse lasers in the axial direction were recorded and simulated, as shown in Figure 10. The fitting values of the M^2^ factor in the horizontal and vertical directions were calculated to be 1.17 and 1.21, respectively, proving the existence of the near-TEM_00_ Gaussian mode in the V-type cavity.

Table 1 compares the pulsed laser performances of the different DQS lasers. We can see from the table that the parameters of the DQS pulsed laser in the experiment are quite satisfactory. It can be concluded from the experimental results that using Au-NBPs grown in a seed-mediated manner as SAs has a good modulation effect on the laser.

## 5. Conclusions

In this paper, we measured the saturable absorption performance of Au-BNPs at a wavelength of ~2.1 µm. By using AOM and Au-BNPs as the saturable absorbers, a DQS Ho:YLF laser, which resulted in the shortest pulse width (54 ns) and the highest peak power (3.87 kW), was realized with an AOM MR of 1 kHz. Compared with the singly Q-switched laser, the DQS laser compressed the pulse width and increased the peak power. The experimental results show that the DQS regime is efficient for pulsed laser operation when running at a wavelength of 2.1 µm.

## Figures and Tables

**Table 1:** A performance comparison of DQS lasers with different 2D saturable absorbers (SAs) at a 2 µm wavelength.

2D SA	Gain Medium	Output Power	Pulse Width	Peak Power	*t_c_*	*P_i_*	Refs
Graphene	Tm:LuAG	-	170 ns	3.12 kW	3.11	97.4	[35]
g-C_3_N_4_	Tm:YAP	274	239 ns	1.15 kW	4.48	241	[36]
WS_2_	Tm:Ca(Gd,Lu)AlO_4_	107	91 ns	1.2 kW	15.38	511.3	[37]
MoS_2_	Tm:Ca(Gd,Lu)AlO_4_	145	82 ns	0.589 kW	9.85	123	[38]
Au-BNPs	Ho:YLF	210	54 ns	3.87 kW	15.2	541.3	This work

## Data Availability

The data presented in this study are available in this article.
